# Supplementary material for: A Critical View of the Application of the APEX Software (Aqueous Photochemistry of Environmentally-Occurring Xenobiotics) to Predict Photoreaction Kinetics in Surface Freshwaters
Source: Molecules. 2019 Dec 18;25(1):9. doi: 10.3390/molecules25010009 (PMC7017383; doi:10.3390/molecules25010009)
Supplement: Supplementary file 1 [file molecules-25-00009-s001.zip › 3.2.4_gcc-4.4.0.zip!/3.2.4_gcc-4.4.0/3.2.4_gcc-4.4.0/doc/octave/html/interpreter/Line_002dOriented-Input.html]

Line-Oriented Input - Untitled


Next: Formatted Output,
Previous: Simple Output,
Up: C-Style I/O Functions


---

#### 14.2.3 Line-Oriented Input

To read from a file it must be opened for reading using `fopen`.
Then a line can be read from the file using `fgetl` as the following
code illustrates

```
     fid = fopen ("free.txt");
     txt = fgetl (fid)
          -| Free Software is needed for Free Science
     fclose (fid);
```

This of course assumes that the file free.txt exists and contains
the line Free Software is needed for Free Science.

— Built-in Function: **fgetl** (fid, len)  
> Read characters from a file, stopping after a newline, or EOF,
> or len characters have been read. The characters read, excluding
> the possible trailing newline, are returned as a string.
>
> If len is omitted, `fgetl` reads until the next newline
> character.
>
> If there are no more characters to read, `fgetl` returns −1.
>
>
>
> **See also:** fread, fscanf.

— Built-in Function: **fgets** (fid, len)  
> Read characters from a file, stopping after a newline, or EOF,
> or len characters have been read. The characters read, including
> the possible trailing newline, are returned as a string.
>
> If len is omitted, `fgets` reads until the next newline
> character.
>
> If there are no more characters to read, `fgets` returns −1.
>
>
>
> **See also:** fputs, fopen, fread, fscanf.
